# Comparing RMR-Derived Proxy Indicators and LEAF-Q for Detecting Low Energy Availability Risk in Mixed-Sport Female Athletes

**DOI:** 10.3390/sports14080319

**Published:** 2026-08-01

**Authors:** Zhi Yun Liu, Wyne Sze Lee, Sonia Kaur Sohi, Xiang Ren Tan, Liyan Huang

**Affiliations:** 1 Health and Social Sciences Cluster, Singapore Institute of Technology, Singapore 828608, Singapore; liu.zhi.yun@sgh.com.sg (Z.Y.L.); lee.wyne.sze@cgh.com.sg (W.S.L.); 2 Sport Science & Sport Medicine, High Performance Sport Institute, Spex Singapore, Singapore 397630, Singapore; soniasohi98@gmail.com; 3 Heat Resilience & Performance Centre, Yong Loo Lin School of Medicine, National University of Singapore, Singapore 117510, Singapore; 4 Human Potential Translational Research Programme, Yong Loo Lin School of Medicine, National University of Singapore, Singapore 117597, Singapore

**Keywords:** energy availability, metabolic suppression, athlete health

## Abstract

Proxy indicators have been proposed as practical screening tools to assess low energy availability (LEA) risk among athletes. We evaluated the utility of the Low Energy Availability in Females Questionnaire (LEAF-Q), resting metabolic rate (RMR) ratio, and relative RMR (rRMR) in identifying LEA risk compared with estimated energy availability (EA). Twenty mixed-sport female athletes (24.8 ± 4.8 years) participated in this cross-sectional study. EA was estimated using four-day food and activity logs. LEAF-Q scores were obtained via questionnaires. RMR was measured via indirect calorimetry while predicted RMR was calculated using the 1980 Cunningham equation. RMR ratio was computed using measured RMR over predicted value, and rRMR computed using measured RMR over fat-free mass (FFM), as assessed by dual-energy X-ray absorptiometry. Participants were classified ‘at risk’ of LEA based on LEAF-Q score ≥8, RMR ratio <0.90, and rRMR <30 kcal/kg FFM/day. RMR-derived measures displayed high sensitivity (100%) and low specificity (44.4%), while LEAF-Q exhibited low sensitivity (27.3%) and moderate specificity (55.6%). EA was moderately correlated with RMR ratio (*r* = 0.47, *p* = 0.035) and strongly correlated with rRMR (*r* = 0.63, *p* = 0.003). RMR-based measures may be promising rapid screening tools for LEA risk in mixed-sport female athletes.

## 1. Introduction

Adequate energy availability (EA) is crucial for maintaining optimal physiological function and performance in athletes [1]. Low energy availability (LEA) is characterised by insufficient residual energy available to support normal physiological processes [2,3]. This condition often arises from inadequate energy intake (EI), increased exercise energy expenditure (EEE), or a combination of both. It can lead to menstrual dysfunction, reduced bone mineral density, hormonal disruptions, and psychological issues, as well as increased risks of injury and illness [1,2,4]. Chronic or severe LEA may also progress to Relative Energy Deficiency in Sport (REDs), a syndrome marked by compromised physiological and psychological functioning [5], thus highlighting the critical need for early detection.

EA can be assessed using a variety of methods, with no single standardised approach. Broadly, these methods can be categorised into laboratory-based assessments, field-based estimation methods, and proxy indicators of LEA [4]. Laboratory-based assessments involve highly controlled measurements of components contributing to EA, such as EI, EEE, and body composition. They generally provide greater precision but are resource-intensive, costly, and difficult to implement in free-living athletes. In contrast, field-based methods estimate EA using self-reported dietary records and exercise logs, offering greater practicality and ecological validity but remaining susceptible to reporting bias and errors in estimating EI and EEE. Finally, proxy indicators of LEA, including questionnaire-based screening tools, physiological markers, and endocrine biomarkers, are commonly used to identify athletes at risk of LEA. However, these indicators reflect the physiological consequences of LEA rather than directly quantifying EA.

Given the logistical challenges associated with laboratory-based assessments, estimated EA derived from dietary and activity records remains one of the most commonly used field-based approaches in LEA research despite its recognised limitations. However, these methods may increase participant burden and require considerable time and resources for data processing [6], potentially limiting the early identification of athletes at risk of LEA. Consequently, there is a need for practical, reliable, and less burdensome approaches to screen for LEA risk. Several proxy indicators have therefore been proposed, including the Low Energy Availability in Females Questionnaire (LEAF-Q) [7], resting metabolic rate (RMR) ratio [8], and relative RMR (rRMR) [9].

The LEAF-Q is a 25-item self-reported questionnaire designed to rapidly detect symptoms associated with LEA, focusing on injury history, gastrointestinal symptoms, and menstrual function [7]. Validated against clinical markers of persistent LEA, the LEAF-Q has demonstrated an acceptable sensitivity (78%) and specificity (90%) among female endurance athletes [7]. Despite potential LEA risk misclassification due to limited generalisability beyond endurance athletes, the LEAF-Q is widely used across endurance and non-endurance sports because of its convenient and rapid administration [10,11].

Alternatively, RMR-derived measures offer more objectivity in assessing LEA risk. RMR represents the body’s basal energy requirements and constitutes approximately 60–75% of total daily energy expenditure [12]. Chronic LEA is often associated with suppressed RMR, where significant reductions up to 20% have been reported, and this is likely attributable to adaptive thermogenesis resulting from prolonged energy deficiency [2]. As a result, the RMR ratio and rRMR have been postulated to identify LEA-related metabolic suppression [13]. However, although RMR-derived measures are increasingly used, their validity as LEA risk screening tools remains less well-established.

The reported global prevalence of LEA in sports ranges from 15% to 88% [14]. This wide variation in prevalence highlights that LEA risk could be influenced by differences in assessment methods, energy expenditure, training volume, sex differences, and sport disciplines. While LEAF-Q has been widely used to detect LEA risk, its validation is largely confined to endurance athletes and there is still a lack of established screening tools for mixed-sport disciplines. Furthermore, a focused lens on female athletes is warranted as they may be more susceptible to LEA than males due to the additional energetic cost of sustaining reproductive function alongside sport-specific demands [2]. Moreover, female athletes are more likely than males to restrict their diet in pursuit of leanness or weight control. Hence, studying female athletes within a mixed-sport cohort is important for evaluating the potential applicability of proxy indicators for identifying LEA risk across different sporting disciplines.

Therefore, our pilot study aimed to evaluate the utility of the LEAF-Q, RMR ratio, and rRMR in identifying LEA risk in mixed-sport female athletes, using estimated EA from four-day food and activity logs as a comparative reference measure. We hypothesised that the RMR ratio would demonstrate greater sensitivity in identifying athletes at risk of LEA and exhibit a stronger correlation with EA than rRMR and LEAF-Q. Identifying accessible and practical screening tools can facilitate the early detection of LEA risk, enabling timely interventions to mitigate detrimental health outcomes.

## 2. Materials and Methods

### 2.1. Participants

Healthy female athletes aged 18 to 39 years from various sports backgrounds (e.g., fencing, rowing, taekwondo) were recruited via word-of-mouth and advertisements at the Singapore Sport Institute and on social media. Participants were excluded if they had a training duration of <12 h per week, injuries requiring training modifications or cessation, contraceptive use within the past year, menstrual disorders affecting period regularity, chronic illnesses or metabolic diseases, use of medications influencing metabolic or reproductive hormones, clinical diagnoses of eating or psychiatric disorders, and current or suspected pregnancy. All participants were screened for eligibility and provided written informed consent before the study commenced. All procedures performed in the study involving human participants were in accordance with the 1964 Declaration of Helsinki and its later amendments or comparable ethical standards. Ethics approval was obtained from the Singapore Sport Institute Institutional Review Board (Ref no.: NU-EXP-029).

### 2.2. Experimental Design

**Figure 1:**
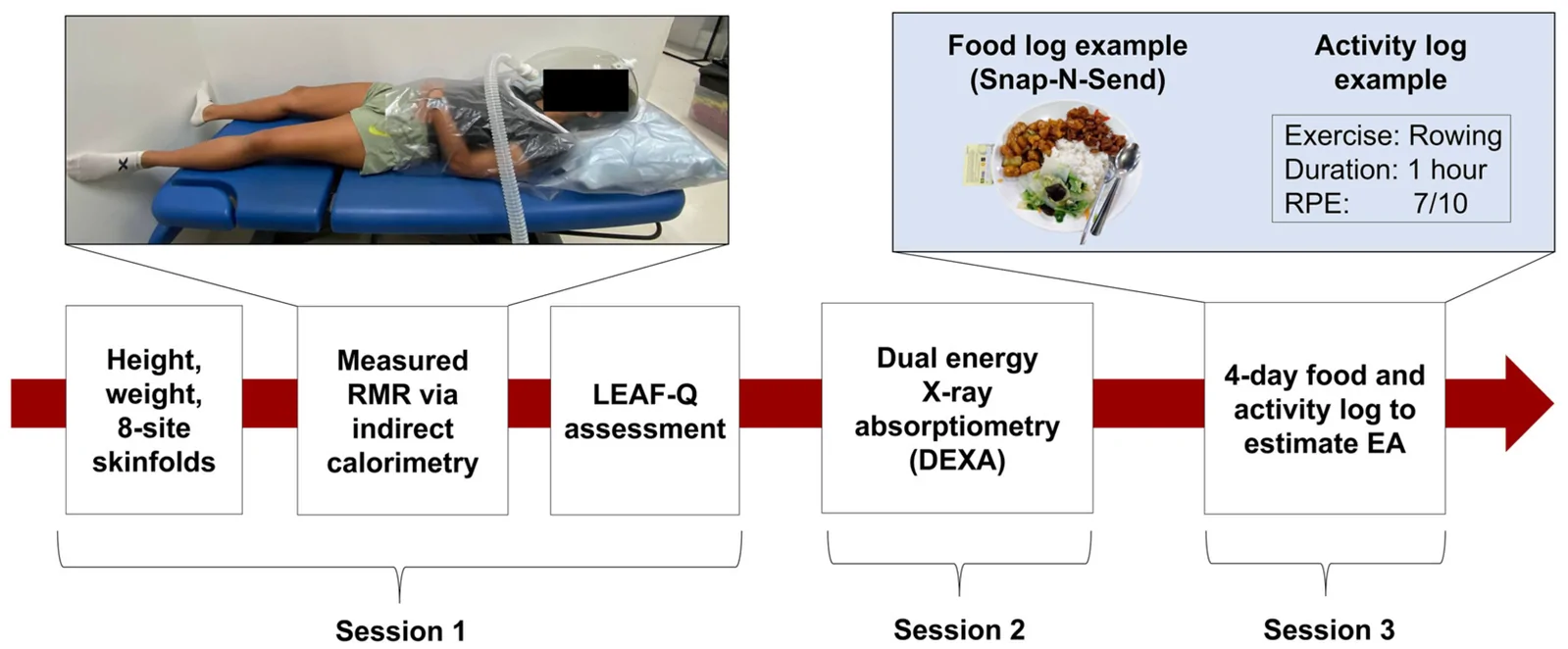
Experimental design involving anthropometry, indirect calorimetry, LEAF-Q, body composition assessments, and food and activity logs over three sessions. Abbreviations: EA = energy availability, LEAF-Q = Low Energy Availability in Females Questionnaire, RMR = resting metabolic rate, RPE = rating of perceived exertion.

The study was conducted as a cross-sectional observational pilot investigation from March to September 2024 to evaluate the feasibility of detecting LEA risk using metabolic and/or questionnaire-based markers for active mixed-sport athletes. In this context, a sample of *n* = 20 athletes was selected, consistent with prior pilot studies on EA and metabolic adaptation studies, which commonly include 6–20 trained participants due to the high commitment (involving self-reporting, food and activity logs) needed to complete the protocols [15,16,17]. All participants completed three data collection sessions (Figure 1). The first session took place at the Singapore Sport Institute, where anthropometric measurements, RMR assessment via indirect calorimetry, and the LEAF-Q were completed. The second session, held within a week after the first session, involved a dual-energy X-ray absorptiometry (DEXA) scan at an external imaging centre to evaluate participants’ body composition. The third session, conducted remotely within two weeks after the first session, required participants to complete a four-day food and activity log to assess EI and EEE, respectively. Participants’ age, sporting background, and average weekly training hours were recorded.

### 2.3. Anthropometry

Participants’ height and weight were measured with a Harpenden wall-mounted stadiometer (Model 602VR, Holtain Ltd., Crymych, Pembrokeshire, Wales, UK) and an electronic weighing scale (Model SPIDER2-150, Mettler Toledo, Greifensee, Switzerland), respectively. Eight-site skinfold measurements (triceps, subscapular, biceps, suprailiac, supraspinale, abdominal, front thigh, and medial calf) were performed using a Harpenden skinfold calliper (Model HSB-BI-3, Baty International, West Sussex, England, UK) by an assessor certified by the International Society for the Advancement of Kinanthropometry (Level 1). The total body mass, fat mass, and lean body mass (LBM) were directly assessed using DEXA, while fat-free mass (FFM) was calculated separately as total mass (kg) − fat mass (kg).

### 2.4. Measurement of RMR

RMR measurements were scheduled to coincide with participants’ habitual wake times. The TrueOne 2400 metabolic cart (Model MMS-2400, ParvoMedics Corp, Salt Lake City, UT, USA) was used in a controlled environment (temperature 21.8–26.9 °C, humidity 62.9–69.1%, barometric pressure 755.7–759.3 mmHg), which was recorded using a data logger (testo 176 P1, Testo SE & Co. KGaA, Titisee-Neustadt, Baden-Württemberg, Germany). Volume and gas calibrations were performed daily according to manufacturer protocols, achieving ±2.9% precision for volume of oxygen consumption (VO_2_) and volume of carbon dioxide production (VCO_2_) measurements. Participants arrived at the laboratory by ride-service vehicles in an overnight fasted state (i.e., no food intake for ≥12 h, no fluid intake for ≥8 h, no caffeine intake for ≥4 h, no alcohol for ≥2.5 h, and no exercise for ≥14 h). This protocol was applied consistently across all participants to standardise pre-test conditions and minimise inter-individual variability arising from recent ingestion or exercise. Participants lay supine on a plinth under a ventilated canopy for 30 min, remaining awake and still during gas exchange measurement. CO_2_ levels in the canopy were maintained between 0.9% and 1.1% using a dilution pump. The first 5 min of data were discarded, and a steady-state period of 3–5 min (≤10% coefficient of variation in VO_2_ and VCO_2_) was selected for RMR determination [18] after visual confirmation on the VO_2_ and VCO_2_ graphs. RMR was computed using the Weir equation [19] via the metabolic cart’s software (TrueOne Metabolic System; RMR 24-3541HU, ParvoMedics Corp, Salt Lake City, UT, USA).

### 2.5. RMR Ratio, rRMR, and LEAF-Q

The 1980 Cunningham equation was used to calculate predicted RMR as follows: Predicted RMR (kcal/day) = 500 + 22 × LBM (kg) [20]. The RMR ratio and rRMR were calculated respectively as follows: (1) RMR ratio = Measured RMR (kcal/day) ÷ Predicted RMR (kcal/day) [13], (2) rRMR = Measured RMR (kcal/day) ÷ FFM (kg) [9]. For LEAF-Q, the paper-based questionnaire was administered and participants’ LEAF-Q scores were tallied using the standardised scoring system [7].

Participants were categorised into ‘At risk’ and ‘Not at risk’ of LEA groups based on their RMR ratio, rRMR values, and LEAF-Q scores. Thresholds for being at risk of LEA were set at RMR ratio <0.90 [13], rRMR <30 kcal/kg FFM/day [9], and LEAF-Q score ≥8 [7].

### 2.6. Measurement of EA

EA was computed using the following equation: EA (kcal/kg FFM/day) = [EI (kcal/day) − EEE (kcal/day)] ÷ FFM (kg). Participants with an estimated EA <30 kcal/kg FFM/day were classified as having LEA based on the commonly applied threshold proposed [3].

EI was evaluated using a food log recorded via the Snap-N-Send method [21]. The log spanned three non-consecutive weekdays and one weekend day, incorporating at least one rest day. Participants photographed every food item with a reference card for scale before and after consumption and provided descriptions of the consumed items, which were submitted via the Telegram messaging application. Ambiguous pictures or descriptions were clarified with the participants. Dietary data were analysed using Foodworks Professional Online (Version 1, Xyris Pty Ltd., Brisbane, Queensland, Australia) to compute EI, with two researchers independently analysing and cross-checking portion sizes to minimise variability.

EEE was assessed over the four days corresponding to the food logs. Participants documented the type and duration of their physical activities, and perceived exertion was rated using the 10-point modified Borg scale [22]. Based on the Compendium of Physical Activities, each activity was assigned a MET value that best matched its type and intensity [23], with ambiguous descriptions clarified to ensure accurate classification. Only planned training activities related to the athlete’s sport were recorded. Corrected MET values were then calculated using participants’ measured RMR to obtain the total thermic effect of activity during exercise [23,24]. The final EEE was calculated by subtracting RMR energy expenditure during the exercise period from total energy expenditure. All data were recorded and processed using Microsoft Excel (Version 2407, Microsoft 365 Apps for Enterprise, Redmond, WA, USA).

### 2.7. Statistical Analysis

Data was analysed using STATA 18.0 Basic Edition (Version 18.0, StataCorp LLC, College Station, TX, USA). Data normality was assessed using the Shapiro–Wilk test. An independent t-test was used to compare the mean EA, RMR ratio, rRMR and LEAF-Q score between LEA and non-LEA groups. Diagnostic 2 × 2 tables were created using the user-written ‘diagti’ package in STATA 18.0 Basic Edition to generate the sensitivity, specificity, and predictive values for each proxy indicator relative to estimated EA. Fisher’s exact test assessed the alignment of the LEAF-Q, RMR ratio, and rRMR risk classifications with estimated EA. Effect sizes were calculated using Cramer’s V and interpreted as small (0.1), medium (0.3), or large (0.5) for values with one degree of freedom [25]. Pearson correlation analysis examined the associations between EA and the proxy indicators. Strength of correlations was interpreted as very weak (<0.20), weak (0.20–0.39), moderate (0.40–0.59), strong (0.60–0.79), or very strong (>0.80) [26]. Goodness-of-fit was assessed using the coefficient of determination (R^2^). A *p*-value of <0.05 was considered statistically significant. Normally distributed data are presented as mean ± SD, while non-normal data are presented as median with interquartile range.

## 3. Results

### 3.1. Participant Characteristics

Of 26 female athletes assessed for eligibility, four were ineligible based on the exclusion criteria (e.g., training <12 h per week and contraceptive use within the past year) and two withdrew before data collection at their coaches’ request, leaving a final sample of 20 participants (mean age 24.8 ± 4.8 years) who were included in the data analysis. Participant characteristics are presented in Table 1. Participants represented various sports, namely artistic gymnastics, badminton, beach volleyball, bowling, canoe/kayak sprint, cycling, fencing, rowing, swimming, taekwondo, and ultimate frisbee. Participants with LEA (*n* = 11) had a lower mean EA (15.3 ± 8.6 vs. 40.1 ± 7.9 kcal/kg FFM/day, *p* < 0.001) and rRMR (25.5 ± 2.5 vs. 29.4 ± 3.9 kcal/kg FFM/day, *p* = 0.016) than those without LEA (*n* = 9). There were no differences in RMR ratio (0.81 ± 0.07 vs. 0.86 ± 0.08, *p* = 0.104) and LEAF-Q scores (5.6 ± 3.9 vs. 7.0 ± 3.5, *p* = 0.430) between both groups.

### 3.2. Diagnostic Performance

Table 2 summarises the diagnostic performance of the proxy indicators. RMR ratio and rRMR demonstrated high sensitivity (100%, 95% CI 71.5–100.0%), identifying all participants classified as having LEA in this cohort, but had low specificity (44.4%, 95% CI 13.7–78.8%) and a positive predictive value of 68.8% (95% CI 41.3–89.0%). The negative predictive value was 100% (95% CI 39.8–100.0%), confirming no false negatives. A large effect size observed (Cramer’s V = 0.6, *p* = 0.026) suggests a stronger association between the RMR-derived measures and estimated LEA classification in this cohort.

In comparison, LEAF-Q displayed low sensitivity (27.3%, 95% CI 6.0–61.0%) and moderate specificity (55.6%, 95% CI 21.2–86.3%), with a positive predictive value of 42.9% (95% CI 9.9–81.6%) and a negative predictive value of 38.5% (95% CI 13.9–68.4%). This indicates a high likelihood of missing true LEA cases. No significant association with LEA was observed (*p* = 0.642).

Although RMR ratio and rRMR exhibited identical diagnostic performance metrics in the present study, they did not classify exactly the same individuals as being at risk of LEA (Table 3). The identical diagnostic performance values arose because the discordant classifications occurred among participants without LEA. Hence, it did not alter the overall sensitivity, specificity, or predictive values.

### 3.3. Associations Between Proxy Indicators and EA

**Figure 2:**
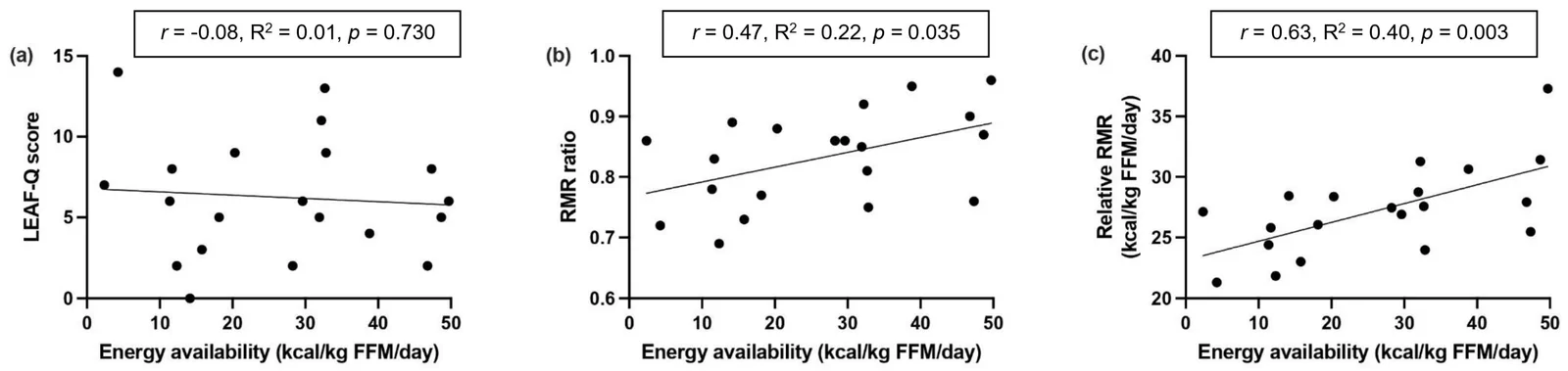
Associations between LEAF-Q score (**a**), RMR ratio (**b**), rRMR (**c**), and EA. Abbreviations: FFM = fat-free mass, LEAF-Q = Low Energy Availability in Females Questionnaire, RMR = resting metabolic rate.

There was negligible association between LEAF-Q and EA (*r* = −0.08, R^2^ = 0.01, *p* = 0.730) (Figure 2a). Conversely, we observed a moderate positive correlation between RMR ratio and EA (*r* = 0.47, R^2^ = 0.22, *p* = 0.035; Figure 2b), and a strong positive correlation for rRMR (*r* = 0.63, R^2^ = 0.40, *p* = 0.003; Figure 2c).

## 4. Discussion

Our findings provide preliminary evidence that RMR-derived measures may be more sensitive than LEAF-Q for the assessment of LEA risk. While both RMR ratio and rRMR exhibited similar diagnostic performances, rRMR was more strongly associated with EA than RMR ratio. This finding was contrary to our hypothesis and could be attributed to the potential advantage of rRMR in capturing subtle inter-individual variations in EA. However, this interpretation remains speculative because rRMR has yet to be validated as a proxy indicator of LEA risk in athletes [5]. To our knowledge, this is the first study to evaluate the utility of rRMR and its correlation with EA, providing preliminary insights regarding its potential application as a screening tool. On the other hand, the findings regarding RMR ratio align with previous research, demonstrating its high sensitivity, low specificity, and positive correlation with EA [27,28].

The observed high sensitivity of RMR-derived measures likely arises from their reliance on direct physiological markers of metabolic suppression. LEA triggers early endocrine adaptations to conserve energy, and these are rapidly reflected in a suppressed RMR [5]. One key mechanism involves cortisol-induced disruption of thyroid hormone levels as part of the body’s adaptive stress response to prolonged energy deficiency [29]. This allows for the detection of metabolic suppression before overt clinical signs such as menstrual dysfunction, recurrent illness, or injury manifest. Therefore, these findings provide preliminary evidence that RMR ratio and rRMR have potential utility as proxy indicators for identifying athletes at risk of LEA. Nonetheless, further validation in larger studies is warranted.

On the other hand, the low specificity observed for both RMR-derived measures could be attributed to inter-individual variability, sport-specific demands, training volume, physical stress, and sleep deprivation, all of which significantly influence metabolic function and RMR [4,5,13]. Recent evidence suggests that menstrual cycle phases exert minimal effect on RMR [9], thus any differences in menstrual phase are unlikely to influence the RMR findings in the present study.

The accuracy of the RMR ratio greatly depends on the predictive equation chosen to compute the predicted RMR, and this could explain the differences in the diagnostic performances between RMR ratio and rRMR. Although the 1980 Cunningham equation was originally developed for non-athletic adults [20], it has been widely adopted in athletic populations due to its strong correlation with measured RMR [28,30,31]. As the key variable in this predictive equation, LBM accounts for roughly 70% of variations in RMR [20]. However, because of this exclusive focus on LBM, it may have contributed to the reduced specificity of the RMR ratio as gender and other anthropometric characteristics contribute to metabolic differences [32]. Notwithstanding these limitations, the 1980 Cunningham equation outperforms other predictive equations and remains the most accurate option for estimating RMR in athletes [30,33,34].

As opposed to relying on predictive equations, adjusting RMR relative to FFM accounts for differences in body composition and potentially enhances the identification of RMR suppression [31]. Unlike RMR ratio, rRMR is calculated entirely using individually measured RMR and FFM. It thereby avoids inaccuracies associated with population-based predictive models and better reflects inter-individual variations, contributing to its observed high sensitivity. Compared with LBM, FFM is the preferred metric as it is more metabolically active and captures a broader and more accurate spectrum of LEA-related metabolic adaptations. This may account for the stronger positive correlation between rRMR and EA as compared to the RMR ratio. However, it should also be acknowledged that this observed correlation may have been partially influenced by mathematical coupling owing to the shared denominator (FFM) in the computations of both rRMR and EA.

Notably, rRMR may display reduced specificity in athletes with higher FFM, increasing the likelihood of false-positive LEA risk classification. This may be because increases in FFM are largely driven by skeletal muscle, which has a lower metabolic rate than high-metabolic-rate organs (e.g., liver, kidneys, heart, and brain) that do not scale proportionally with FFM [35]. Consequently, rRMR may appear lower in athletes with high FFM despite adequate EA. In addition, FFM may fluctuate across the training season and is typically higher during competitive periods than the off-season, further complicating interpretation of rRMR values [35]. To minimise false positives, rRMR may be assessed during non-competitive periods or interpreted relative to an individual’s baseline rather than an absolute threshold. However, these suggestions remain to be further investigated in future studies.

In comparison to RMR-derived measures, LEAF-Q displayed notably lower sensitivity (27.3%) and specificity (55.6%) in this mixed-sport cohort. Our findings are corroborated by previous studies involving similar cohorts where the LEAF-Q showed limited specificity [36,37]. This contrasts with studies conducted in endurance athletes, where sensitivity and specificity values observed ranged from 78–79% and 50–90% [7,38]. The reduced diagnostic performance and negligible association with EA in this study may be attributed to the low relevance of questions regarding injuries and gastrointestinal symptoms to mixed-sport athletes. For instance, overuse injuries are commonly linked to endurance sports as a direct result of LEA [39]. However, such injuries can also occur in non-endurance, high-impact sports (e.g., fencing, gymnastics) due to the nature of the sport itself, with physical and mechanical demands contributing to injury risk [40,41]. Furthermore, gastrointestinal symptoms are more prevalent among endurance athletes, possibly owing to exercise-induced gut damage caused by high-volume training [5,37]. Hence, the digestive issues reported in the LEAF-Q may be less pertinent to athletes in other sports with lower physical demands and training volume (for instance, bowling). Collectively, these factors may affect the LEAF-Q scoring accuracy in non-endurance athletes, increasing the risk of misclassification when applied to mixed-sport cohorts.

An additional consideration is the potential influence of the study’s exclusion criteria on the apparent diagnostic performance of the LEAF-Q. We have excluded participants with injuries requiring training modifications or cessation, recent contraceptive use, or menstrual disorders affecting cycle regularity, since these individuals may exhibit clinical features captured by the LEAF-Q (e.g., injury history, contraceptive use, menstrual dysfunction, and gastrointestinal symptoms) independently of LEA. Nonetheless, this exclusion may have limited the generalisability of our results to athletes with such conditions, and the performance of the LEAF-Q in these populations warrants further investigation. Overall, our findings suggest that the LEAF-Q demonstrated poorer diagnostic performance in this mixed-sport cohort and may have limited utility for identifying LEA risk among female athletes from diverse sports.

This study recruited athletes from a range of sporting disciplines with differing training loads, energy demands, body composition profiles, and injury patterns. These variables may have influenced the performance of the proxy indicators in detecting LEA risk. Nevertheless, such heterogeneity was necessary to evaluate the broader utility of these proxy indicators regardless of sporting background.

Prolonged LEA is detrimental to an athlete’s health and performance. Thus, highly sensitive screening tools are necessary to facilitate early detection despite a risk of false positives. While false positives may cause unnecessary treatment and psychological distress, false negatives can instead delay the identification and management of LEA, hence worsening outcomes. Therefore, such tools are prioritised to mitigate the greater risks posed by false negatives. In this cohort, both the RMR ratio and rRMR demonstrated high sensitivity despite their modest specificity, while the LEAF-Q displayed poor sensitivity. Hence, RMR ratio and rRMR may serve as promising screening tools, with athletes flagged as ‘at risk’ of LEA referred to a sports medicine physician for further assessment.

There are some limitations to note in this study. The small sample size and cross-sectional study design may limit the generalisability of our results to the broader athletic population. Therefore, future studies should include larger sample sizes from various sports to yield more representative findings. Additionally, while we have adopted current best practices for the indirect calorimetry protocol, it was challenging to objectively ascertain whether all participants were fully rested and relaxed before and during the RMR measurements. This may have hindered the attainment of steady-state conditions and influenced the RMR values obtained. Future research can strive to improve measurement accuracy by monitoring physiological parameters (e.g., heart rate variability) to confirm a rested state, as well as verifying the sleep quality and quantity of participants through sleep-tracking devices prior to RMR testing. Furthermore, the duration of sport participation and training age were not recorded in this study. As training history may influence physiological adaptations, energy requirements, and susceptibility to LEA, the absence of this data may have introduced unaccounted variability into the findings. While it is important to understand sport-specific differences in LEA risk detection across the tools, such comparisons were not made in this study due to the relatively small sample size and limited representation from each sport. Lastly, information regarding postpartum status, breastfeeding, and motherhood, which was not recorded in this study, should be considered in future research as these may independently influence RMR, EA, and LEAF-Q responses.

Estimated EA was derived from four-day food and activity logs and therefore represents only a short-term snapshot of participants’ energy status. In contrast, suppressed RMR may reflect a longer-term physiological adaptation to sustained LEA. Consequently, a temporal mismatch may exist between the EA classification and RMR-derived measures, potentially influencing the observed diagnostic performance. Moreover, both EI and EEE values derived from self-reported data are subject to potential reporting biases and estimation errors, making it challenging to determine EA accurately. This also impacts the diagnostic performances interpreted in this study. Future studies may benefit from using longer-duration (up to 7 days) or repeated assessments of dietary intake and physical activity to better characterise habitual EA and its relationship with RMR-derived measures. The use of more objective measures, such as weighed food records and the use of wearable activity trackers, may also improve the accuracy of EI and EEE assessment.

Lastly, while DEXA is highly accurate for determining body composition, its high cost and limited accessibility restrict its use in most applied settings and hence limit the adoption of RMR-derived proxy indicators. Therefore, exploring alternative methods such as skinfold measurements and bioimpedance analysis may offer a more affordable and accessible avenue for LEA risk screening in larger athletic populations.

## 5. Conclusions

In summary, the present findings suggest that the RMR ratio and rRMR are promising tools for assessing LEA risk and may be more sensitive than the LEAF-Q in a cohort of mixed-sport female athletes. However, sport-specific comparisons are not possible given the relatively small sample size of this study. Future research with larger and more diverse cohorts should incorporate more objective parameters to ensure the accuracy of EI, EEE, and RMR assessments. Exploring affordable and accessible tools for body composition assessment may also enhance the practicality of using RMR-derived measures in applied settings. This study provides preliminary evidence supporting the usefulness of RMR-derived proxy indicators as screening tools for LEA risk in real-world settings. These tools may help identify athletes who could benefit from further assessment for LEA, particularly where appropriate equipment and expertise are available.

## Figures and Tables

**Table 1:** Participant details categorised by LEA classification.

	All (*n* = 20)	Non-LEA (*n* = 9)	LEA (*n* = 11)
*Characteristics*			
Age (years)	24.8 ± 4.8	22.9 ± 4.4	26.5 ± 4.7
Weight (kg)	56.7 ± 6.1	52.9 ± 6.6	59.7 ± 3.6
Height (cm)	161.7 ± 7.4	159.5 ± 9.3	163.5 ± 5.0
Body mass index (kg/m^2^)	21.6 ± 1.6	20.7 ± 1.5	22.4 ± 1.3
Average training (h/week)	15.0 (13.0–21.5)	14.0 (12.5–15.0)	19.0 (14.5–24.0)
*Body composition—skinfolds*			
Sum of 8 skinfolds (mm)	101.8 ± 25.0	102.8 ± 22.8	101.0 ± 27.8
*Body composition—DEXA*			
Fat mass (kg)	14.1 ± 2.7	14.0 ± 2.7	14.1 ± 2.9
Body fat (%)	24.9 ± 5.2	26.8 ± 5.3	23.4 ± 4.7
FFM (kg)	42.9 ± 6.6	38.8 ± 6.5	46.3 ± 4.5
FFM (%)	75.1 ± 5.2	73.2 ± 5.3	76.6 ± 4.7
LBM (kg)	40.5 ± 6.3	36.6 ± 6.3	43.8 ± 4.3
LBM (%)	70.9 ± 5.1	69.0 ± 5.4	72.5 ± 4.5
*Variables*			
EI (kcal/kg FFM/day)	44.7 ± 12.8	54.9 ± 8.0	36.4 ± 9.6
EEE (kcal/kg FFM/day)	17.2 (12.6–20.1)	11.0 (9.9–17.5)	17.9 (15.6–29.9)
EA (kcal/kg FFM/day)	26.5 ± 15.0	40.1 ± 7.9	15.3 ± 8.6 *
Measured RMR (kcal/day)	1152 ± 118	1124 ± 134	1175 ± 104
Predicted RMR (kcal/day)	1392 ± 138	1306 ± 138	1463 ± 94
*LEA risk proxy indicators*			
RMR ratio	0.83 ± 0.08	0.86 ± 0.08	0.81 ± 0.07
rRMR (kcal/kg FFM/day)	27.3 ± 3.7	29.4 ± 3.9	25.5 ± 2.5 *
LEAF-Q score	6.3 ± 3.7	7.0 ± 3.5	5.6 ± 3.9

Note: Normally distributed data are presented as mean ± SD and non-normal data as median with interquartile range in parentheses in this table. * denotes significance set at *p* < 0.05. Abbreviations: DEXA = dual-energy X-ray absorptiometry, EA = energy availability, EEE = exercise energy expenditure, EI = energy intake, FFM = fat-free mass, LBM = lean body mass, LEA = low energy availability, LEAF-Q = Low Energy Availability in Females Questionnaire, RMR = resting metabolic rate, rRMR = relative RMR.

**Table 2:** Diagnostic performance of the proxy indicators in detecting LEA risk.

Proxy Indicator (Threshold)	LEAF-Q (≥8)	RMR Ratio (<0.90)	rRMR (<30 kcal/kg FFM/day)
Sensitivity	27.3%	100.0%	100.0%
[95% CI]	[6.0%, 61.0%]	[71.5%, 100.0%]	[71.5%, 100.0%]
Specificity	55.6%	44.4%	44.4%
[95% CI]	[21.2%, 86.3%]	[13.7%, 78.8%]	[13.7%, 78.8%]
Positive predictive value	42.9%	68.8%	68.8%
[95% CI]	[9.9%, 81.6%]	[41.3%, 89.0%]	[41.3%, 89.0%]
Negative predictive value	38.5%	100.0%	100.0%
[95% CI]	[13.9%, 68.4%]	[39.8%, 100.0%]	[39.8%, 100.0%]
*p*-value ^†^	0.642	0.026 *	0.026 *

* denotes significance set at *p* < 0.05. ^†^ Each proxy indicator’s classification alignment with estimated EA was assessed via Fisher’s exact test. Abbreviations: CI = confidence interval, LEAF-Q = Low Energy Availability in Females Questionnaire, RMR = resting metabolic rate, rRMR = relative RMR.

**Table 3:** Mapping of LEA and LEA risk according to participant.

Participant	EA	LEAF-Q	RMR Ratio	rRMR
1	▲		●	●
2	▲	●	●	●
3	▲		●	●
4	▲		●	●
5	▲	●	●	●
6	▲		●	●
7	▲		●	●
8	▲		●	●
9	▲		●	●
10	▲	●	●	●
11	▲		●	●
12		●	●	●
13		●		
14				
15			●	
16			●	●
17				
18		●	●	●
19		●	●	●
20				●

Legend: ▲ = LEA derived from estimated EA <30 kcal/kg FFM/day, ● = at risk of LEA based on thresholds. Abbreviations: EA = energy availability, LEAF-Q = Low Energy Availability in Females Questionnaire, RMR = resting metabolic rate, rRMR = relative RMR.

## Data Availability

The raw data supporting the conclusions of this article will be made available by the corresponding authors upon reasonable request.

## Abbreviations

The following abbreviations are used in this manuscript:

DEXA	Dual-Energy X-ray Absorptiometry
EA	Energy Availability
EI	Energy Intake
EEE	Exercise Energy Expenditure
FFM	Fat-Free Mass
LBM	Lean Body Mass
LEA	Low Energy Availability
LEAF-Q	Low Energy Availability in Females Questionnaire
REDs	Relative Energy Deficiency in Sport
rRMR	Relative Resting Metabolic Rate
RMR	Resting Metabolic Rate
VCO_2_	Volume of Carbon Dioxide Production
VO_2_	Volume of Oxygen Consumption

## References

[1] Melin A.K., Areta J.L., Heikura I.A., Stellingwerff T., Torstveit M.K., Hackney A.C. (2024). Direct and indirect impact of low energy availability on sports performance. Scand. J. Med. Sci. Sports.

[2] Areta J.L., Taylor H.L., Koehler K. (2021). Low energy availability: History, definition and evidence of its endocrine, metabolic and physiological effects in prospective studies in females and males. Eur. J. Appl. Physiol..

[3] Melin A.K., Heikura I.A., Tenforde A., Mountjoy M. (2019). Energy availability in athletics: Health, performance, and physique. Int. J. Sport Nutr. Exerc. Metab..

[4] Kuikman M.A., Coates A.M., Burr J.F. (2022). Markers of low energy availability in overreached athletes: A systematic review and meta-analysis. Sports Med..

[5] Mountjoy M., Ackerman K.E., Bailey D.M., Burke L.M., Constantini N., Hackney A.C., Heikura I.A., Melin A., Pensgaard A.M., Stellingwerff T. (2024). 2023 International Olympic Committee’s (IOC) consensus statement on relative energy deficiency in sport (REDs). Br. J. Sports Med..

[6] Burke L.M., Lundy B., Fahrenholtz I.L., Melin A.K. (2018). Pitfalls of conducting and interpreting estimates of energy availability in free-living athletes. Int. J. Sport Nutr. Exerc. Metab..

[7] Melin A., Tornberg Å.B., Skouby S., Faber J., Ritz C., Sjödin A., Sundgot-Borgen J. (2014). The LEAF questionnaire: A screening tool for the identification of female athletes at risk for the female athlete triad. Br. J. Sports Med..

[8] De Souza M.J., Hontscharuk R., Olmsted M., Kerr G., Williams N.I. (2007). Drive for thinness score is a proxy indicator of energy deficiency in exercising women. Appetite.

[9] Kuikman M.A., McKay A.K.A., Minahan C., Harris R., Elliott-Sale K.J., Stellingwerff T., Smith E.S., McCormick R., Tee N., Skinner J. (2024). Effect of menstrual cycle phase and hormonal contraceptives on resting metabolic rate and body composition. Int. J. Sport Nutr. Exerc. Metab..

[10] Magee M.K., Jones M.T., Fields J.B., Kresta J., Khurelbaatar C., Dodge C., Merfeld B., Ambrosius A., Carpenter M., Jagim A.R. (2023). Body composition, energy availability, risk of eating disorder, and sport nutrition knowledge in young athletes. Nutrients.

[11] Witkoś J., Błażejewski G., Gierach M. (2022). An assessment of the early symptoms of energy deficiency as a female athlete triad risk among the Polish national kayaking team using LEAF-Q. Int. J. Environ. Res. Public Health.

[12] Watson A.D., Zabriskie H.A., Witherbee K.E., Sulavik A., Gieske B.T., Kerksick C.M. (2019). Determining a resting metabolic rate prediction equation for collegiate female athletes. J. Strength Cond. Res..

[13] Sterringer T., Larson-Meyer D.E. (2022). RMR ratio as a surrogate marker for low energy availability. Curr. Nutr. Rep..

[14] Gallant T.L., Ong L.F., Wong L., Sparks M., Wilson E., Puglisi J.L., Gerriets V.A. (2025). Low energy availability and relative energy deficiency in sport: A systematic review and meta-analysis. Sports Med..

[15] Lee S., Kuniko M., Han S., Oh T., Taguchi M. (2020). Association of low energy availability and suppressed metabolic status in Korean male collegiate soccer players: A pilot study. Am. J. Men’s Health.

[16] Pritchett K., DiFolco A., Glasgow S., Pritchett R., Williams K., Stellingwerff T., Roney P., Scaroni S., Broad E. (2021). Risk of low energy availability in national and international level paralympic athletes: An exploratory investigation. Nutrients.

[17] Taguchi M., Moto K., Lee S., Torii S., Hongu N. (2020). Energy intake deficiency promotes bone resorption and energy metabolism suppression in Japanese male endurance runners: A pilot study. Am. J. Men’s Health.

[18] Fullmer S., Benson-Davies S., Earthman C.P., Frankenfield D.C., Gradwell E., Lee P.S.P., Piemonte T., Trabulsi J. (2015). Evidence analysis library review of best practices for performing indirect calorimetry in healthy and non–critically ill individuals. J. Acad. Nutr. Diet..

[19] Weir J.B.d.V. (1949). New methods for calculating metabolic rate with special reference to protein metabolism. J. Physiol..

[20] Cunningham J.J. (1980). A reanalysis of the factors influencing basal metabolic rate in normal adults. Am. J. Clin. Nutr..

[21] Costello N., Deighton K., Dyson J., McKenna J., Jones B. (2017). Snap-N-Send: A valid and reliable method for assessing the energy intake of elite adolescent athletes. Eur. J. Sport Sci..

[22] Heikura I.A., Uusitalo A.L.T., Stellingwerff T., Bergland D., Mero A.A., Burke L.M. (2018). Low energy availability is difficult to assess but outcomes have large impact on bone injury rates in elite distance athletes. Int. J. Sport Nutr. Exerc. Metab..

[23] Ainsworth B.E., Haskell W.L., Herrmann S.D., Meckes N., Bassett D.R., Tudor-Locke C., Greer J.L., Vezina J., Whitt-Glover M.C., Leon A.S. (2013). 2011 compendium of physical activities: A second update of codes and MET values. Med. Sci. Sports Exerc..

[24] Heydenreich J., Schutz Y., Melzer K., Kayser B. (2019). Comparison of conventional and individualized 1-MET values for expressing maximum aerobic metabolic rate and habitual activity related energy expenditure. Nutrients.

[25] Cohen J. (2013). Statistical Power Analysis for the Behavioral Sciences.

[26] Evans J.D. (1996). Straightforward Statistics for the Behavioral Sciences.

[27] Staal S., Sjödin A., Fahrenholtz I., Bonnesen K., Melin A.K. (2018). Low RMR ratio as a surrogate marker for energy deficiency, the choice of predictive equation vital for correctly identifying male and female ballet dancers at risk. Int. J. Sport Nutr. Exerc. Metab..

[28] Strock N.C.A., Koltun K.J., Southmayd E.A., Williams N.I., De Souza M.J. (2020). Indices of resting metabolic rate accurately reflect energy deficiency in exercising women. Int. J. Sport Nutr. Exerc. Metab..

[29] Hackney A.C., Moore S.R., Smith-Ryan A. (2025). Low energy availability, carbohydrate intake, and relative energy deficiency in sport: The low triiodothyronine hypothesis. Int. J. Sports Physiol. Perform..

[30] Jagim A.R., Camic C.L., Kisiolek J., Luedke J., Erickson J., Jones M.T., Oliver J.M. (2018). Accuracy of resting metabolic rate prediction equations in athletes. J. Strength Cond. Res..

[31] O’Neill J.E.R., Corish C.A., Horner K. (2023). Accuracy of resting metabolic rate prediction equations in athletes: A systematic review with meta-analysis. Sports Med..

[32] Jagim A.R., Jones M.T., Askow A.T., Luedke J., Erickson J.L., Fields J.B., Kerksick C.M. (2023). Sex differences in resting metabolic rate among athletes and association with body composition parameters: A follow-up investigation. J. Funct. Morphol. Kinesiol..

[33] Jagim A.R., Camic C.L., Askow A., Luedke J., Erickson J., Kerksick C.M., Jones M.T., Oliver J.M. (2019). Sex differences in resting metabolic rate among athletes. J. Strength Cond. Res..

[34] ten Haaf T., Weijs P.J.M. (2014). Resting energy expenditure prediction in recreational athletes of 18–35 years: Confirmation of Cunningham equation and an improved weight-based alternative. PLoS ONE.

[35] Kuikman M.A., Smith E., McKay A.K.A., McCormick R., Ackerman K.E., Harris R., Elliott-Sale K.J., Stellingwerff T., Burke L.M. (2025). Impact of acute dietary and exercise manipulation on next-day RMR measurements and DXA body composition estimates. Med. Sci. Sports Exerc..

[36] Appaneal R., Burke L., Drew M., Hughes D., Lovell G., Lundy B., Rogers M., Vlahovich N., Waddington G. (2021). The diagnostic performance of the Low Energy Availability in Females Questionnaire (LEAF-Q) in a mixed-sport cohort. J. Sci. Med. Sport.

[37] Rogers M.A., Drew M.K., Appaneal R., Lovell G., Lundy B., Hughes D., Vlahovich N., Waddington G., Burke L.M. (2021). The utility of the low energy availability in females questionnaire to detect markers consistent with low energy availability-related conditions in a mixed-sport cohort. Int. J. Sport Nutr. Exerc. Metab..

[38] Wasserfurth P., Halioua R., Toepffer D., Lautz Z., Engel H., Melin A.K., Torstveit M.K., Claussen M.C., Koehler K. (2025). Screening for Relative Energy Deficiency in Sport: Detection of clinical indicators in female endurance athletes. Med. Sci. Sports Exerc..

[39] Dasa M.S., Friborg O., Kristoffersen M., Pettersen G., Sagen J.V., Sundgot-Borgen J., Rosenvinge J.H. (2023). Evaluating the suitability of the Low Energy Availability in Females Questionnaire (LEAF-Q) for female football players. Sports Med. Open.

[40] Moss S.L., Randell R.K., Burgess D., Ridley S., Cairealláin C.Ó., Allison R., Rollo I. (2021). Assessment of energy availability and associated risk factors in professional female soccer players. Eur. J. Sport Sci..

[41] Vardardottir B., Gudmundsdottir S.L., Tryggvadottir E.A., Olafsdottir A.S. (2024). Patterns of energy availability and carbohydrate intake differentiate between adaptable and problematic low energy availability in female athletes. Front. Sports Act. Living.

